# β-ecdysone/PLGA composite scaffolds promote skull defect healing in diabetic rat

**DOI:** 10.3389/fbioe.2024.1536102

**Published:** 2025-01-13

**Authors:** Yicai Luo, Ziwei Wu, Yingjuan Zhang, Yang Qiao, Yinge Wei, Xuan Yan, Xiangyu Ma, Xianxian Huang, Xiaoxia Zhong, Zhimao Ye, Xinping Lu, Hongbing Liao

**Affiliations:** Guangxi Key Laboratory of Oral and Maxillofacial Rehabilitation and Reconstruction, College and Hospital of Stomatology, Guangxi Medical University, Nanning, Guangxi, China

**Keywords:** diabetes, β-ecdysone, PLGA, tissue engineering scaffold, diabetic rat skull defect model

## Abstract

**Introduction:**

Diabetes mellitus often leads to bone metabolism disorders, hindering bone regeneration and delaying the healing of bone defects. β-Ecdysone, a plant-derived hormone known for its wide range of physiological activities, possesses hypoglycemic effects and promotes osteogenic differentiation. This study developed a composite PLGA slow-release scaffold loaded with β-ecdysone to enhance its bioavailability through topical administration and to investigate its potential to heal diabetic bone defects.

**Methods:**

The composite scaffolds were fabricated using solution casting/particle leaching and freeze-drying techniques. Then a series of characterizations were subjected to test the performance of composite scaffolds, and *in vitro* safety of the composite scaffolds was tested by CCK8 assay and live/dead cell staining. Further, micro-CT and histology to evaluate the effect of β-E/PLGA composite scaffolds on healing of skull defects in diabetic rats at 4 and 8 weeks after implantation. Simultaneously, the safety of the scaffolds *in vivo* was also evaluated.

**Results:**

The material characterization results indicated that, in comparison to the single-pore size scaffold, the composite scaffold exhibited superior porosity, swelling ratio, drug loading capacity, and mechanical properties. Additionally, the composite scaffolds showed appropriate degradation performance and sustained drug release profiles. The CCK8 cytotoxicity assay and live/dead cell staining demonstrated that BMSCs survived and proliferated on the composite scaffold under both low-glucose and high-glucose conditions. Micro-CT and histological investigation demonstrated that β-E/PLGA composite scaffolds promoted new bone growth in the skull defect region of diabetic rats.

**Conclusion:**

Overall, these findings suggest that the β-E/PLGA composite scaffolds promote the healing of bone defects in diabetic rats. The combination of β-ecdysone and tissue-engineered scaffolds presents a promising approach for treating diabetes-related bone defects.

## 1 Introduction

Diabetes mellitus is a widespread chronic metabolic disorder marked by sustained hyperglycemia. Prolonged elevated blood sugar levels can cause damage to multiple organs, with osteoporosis being one of the common complications. Research has demonstrated that persistent hyperglycemia significantly delays the healing of bone defects compared to individuals with normal blood glucose levels (Preshaw et al., 2012). Hyperglycemia can lead to reduced proliferation and premature apoptosis of bone marrow mesenchymal stem cells (BMSCs), thereby inhibiting osteogenesis (Li et al., 2021). β-Ecdysone, a hormone structurally similar to estradiol, plays a role in regulating the molting process in insects. It is widely distributed across various plant species, including hyssop, Kawakawa hyssop, Dewy’s weed, as well as ferns like Purple Minnow (Das et al., 2021). β-Ecdysone shows a range of physiological activities, effectively promoting nucleic acid, carbohydrate, and lipid metabolism, as well as protein synthesis in various tissues and organs. It also possesses anti-inflammatory, anti-tumor, and antioxidant properties (Parr et al., 2014; Xu et al., 2018; Wang et al., 2020). Studies have shown that β-Ecdysone not only improved hyperglycemia in streptozotocin-induced diabetic gerbils (Mallek et al., 2018), but also lowered blood sugar levels in diet-induced obese mice (Kizelsztein et al., 2009). In terms of osteogenesis, β-Ecdysone induces osteogenic differentiation of bone marrow mesenchymal stem cells in mice, effectively alleviating osteoporosis symptoms (Gao et al., 2008).

Generally, drugs with low water solubility affects its bioavailability in the body when administered systemically. Conversely, topical drug application is an effective strategy to enhance drug bioavailability (Kulchar et al., 2023). Local drug delivery systems made from biodegradable materials, such as microspheres and scaffolds, are commonly used for topical drug application. These systems allow relative control over drug release, enhancing bioavailability and protecting the drug from degradation, thus prolonging its activity. Poly (lactic-co-glycolic acid) (PLGA), a biodegradable polymer formed by the random polymerization of lactic acid and glycolic acid monomers, is widely used in pharmaceuticals and biomedical engineering due to its excellent biocompatibility (Zhao et al., 2021). PLGA also functions as a localized drug delivery carrier, enabling precise drug release and increasing local drug concentration (Xiao et al., 2021). It creates a temporary matrix that supports new tissue development and promotes the regeneration of tissue defects (Perez and Mestres, 2016). With its improved biodegradability and biocompatibility, PLGA is particularly well-suited for repairing bone defects (Danhier et al., 2012).

In this study, composite PLGA slow-release scaffolds were first prepared, and their physical properties and safety were verified. The effects of β-E/PLGA composite scaffolds on the healing of cranial bone defects in diabetic rats were then evaluated through *in vivo* experiments, to provide new clinical insights for the treatment of diabetic bone defects.

## 2 Materials and methods

### 2.1 Materials

β-Ecdysone (purity ≥98%) was sourced from MCE Biotechnology (New Jersey, United States), while PLGA (lactide-to-glycolide molar ratio 50:50, MW 160,000) was supplied by Daigang Biotechnology Co., Ltd. (Shandong, China). Polyethylene glycol (PEG) and dichloromethane were acquired from Shanghai Aladdin Biochemical Technology Co., Ltd. (Shanghai, China). The CCK8 kit was obtained from Guangzhou Saiguo Biotechnology Co., Ltd. (Guangzhou, China) and Live/Dead Staining Kit were provided by Shanghai Biyuntian Biotechnology Co., Ltd. (Shanghai, China). Streptozotocin was supplied by Shanghai McLean Biochemical Technology Co., Ltd. (Shanghai, China). Simulated body fluid (SBF), Hematoxylin and Eosin (H&E) staining kit, and Masson’s trichrome staining kit were obtained from Beijing Solepol Science and Technology Co., Ltd. (Beijing, China). RUNX2 antibody was sourced from Wuhan Doctoral Bioengineering Co., Ltd. (Wuhan, China).

### 2.2 Preparation of the β-E/PLGA composite scaffolds

**FIGURE 1:**
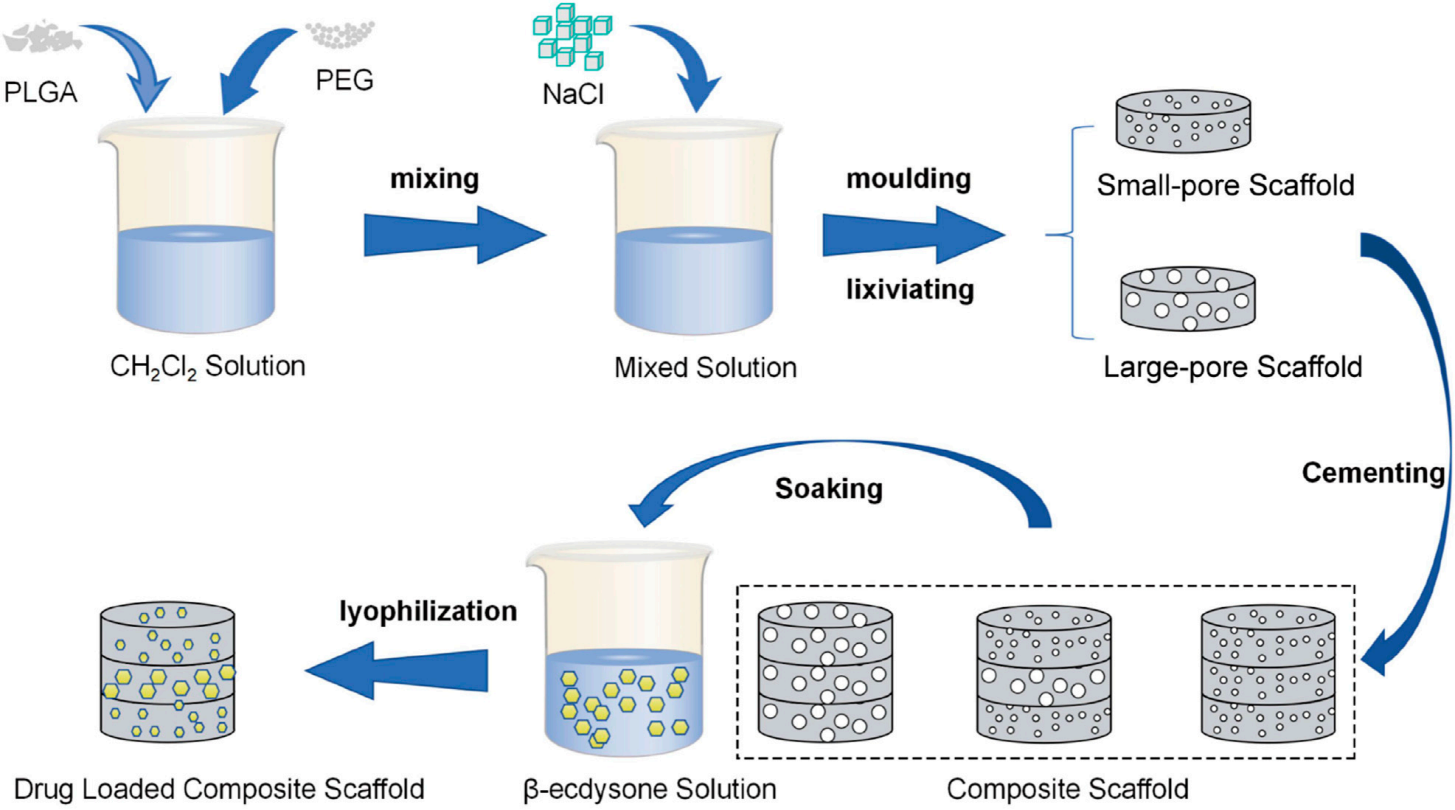
Flow chart representing the preparation of β-E/PLGA composite scaffolds.

PLGA scaffolds were fabricated using the solution casting/particle leaching method. Sodium chloride (NaCl) of varying particle sizes (large: 250–400 μm, small: 100–250 μm) was selected as the porogen. Small-pore, large-pore, and composite scaffolds were each prepared using this method. Polyethylene glycol (PEG), known for its excellent aqueous solubility, biocompatibility, and safety, was also used alongside NaCl as a porogen to enhance the pore connectivity of the PLGA scaffolds (Thadavirul et al., 2014). To prepare the scaffolds, a 10% w/v solution of PLGA was first created by dissolving a specific weight of PLGA and PEG (in a 1:1 ratio) in dichloromethane. Sodium chloride (NaCl) of various particle sizes was screened and added to the PLGA solution at a ratio of 1: 15 (PLGA: NaCl), followed by thorough mixing. This mixture was then poured into a circular mold with a diameter of 5 mm and a thickness of 0.5 mm, yielding two types of monolayer PLGA modules with different pore sizes. The monolayer modules were bonded together using methylene chloride as an adhesive. As a result, large-pore-size scaffolds (S1), small-pore-size scaffolds (S2), and composite scaffolds (S3) were prepared. The bonded modules were then soaked in deionized water for 3 days, with the water replaced every 8 h. After the porogen was fully dissolved, the modules were freeze-dried for 24 h. To fabricate the drug-loaded scaffolds, a 1% w/v β-Ecdysone solution was prepared, and the scaffolds were soaked in this solution for 24 h before being freeze-dried. The specific procedure is illustrated in Figure 1.

### 2.3 Characterization of the PLGA composite scaffolds

#### 2.3.1 Porosity

The porosity of the scaffold is measured using the liquid displacement method, with ethanol as the displacement liquid, and at least three parallel samples are set for each group. The initial weight of each group of stents was recorded as (m_0_). A graduated measuring cylinder was filled with 2 mL of anhydrous ethanol, and a stent with a diameter of 5 mm and a thickness of 1.5 mm was immersed in the liquid. The cylinder was sealed with a film, and the volume of the stent was recorded as (V). Small holes were then created in the sealing membrane using a sterile syringe needle, and the stent was subjected to a vacuum of −0.1 MPa until it was fully submerged. At this point, it was assumed that the pores in the stent were filled with anhydrous ethanol. The stents were quickly removed from the measuring cylinder using disposable sterile tweezers, and their final weight was recorded as (m_1_) after any excess liquid was blotted away with filter paper. The porosity was then calculated using the following formula:

$$\text{Porosity}=\frac{m_{1}-m_{0}}{\rho \times V}\times 100\%$$

#### 2.3.2 Swelling ratio

The swelling behavior of the prepared stents was analyzed by detecting the weight change of the stent samples before and after FBS immersion. In brief, the initial weight of the stent before immersion was recorded as (m_0_). After immersing the stent in FBS solution for 24 h, the sample was removed, weighed, and recorded as (m_1_). The calculation formula is as follows:

$$\text{Swelling ratio}=\frac{\left(m_{1}-m_{0}\right)}{m_{0}}\times 100\%$$

#### 2.3.3 Drug content

The weight of a lyophilized carrier support weighing 10 mg was referred to as (m_0_). Then, 1 mL of acetonitrile and 2 mL of FBS solution were added. After the carrier support was completely dissolved, the absorbance was measured under UV light at a wavelength of 275 nm. The content of β-Ecdysone in (m_0_) was calculated using a previously established standard curve and recorded as (m_1_). The calculation formula is as follows:

$$\text{Drug content}=\frac{m_{1}}{m_{0}}\times 100\%$$

#### 2.3.4 Compressive strength

The large aperture bracket, small aperture bracket, and composite aperture bracket (diameter 5 mm, height 1.5 mm) were selected for compressive strength testing using an electronic universal material testing machine (AGX-10kNVD, SHIMADZU, Japan). To ensure proper contact, the parallelism of each plane was maintained, allowing the bracket’s surface to make full contact with the probe. During the test, the probe applied axial compression to the sample holder at a rate of 0.5 mm/min, with a maximum stroke of 1 mm. Displacement data were recorded throughout the loading process to generate curves, and three samples from each group were tested.

#### 2.3.5 Fourier transform infrared spectroscopy

To determine whether β-Ecdysone was successfully incorporated into the PLGA scaffolds, Fourier transform infrared (FTIR) spectroscopy was employed to analyze changes in the functional groups of the drug-loaded composite scaffolds. The β-E/PLGA scaffolds, PLGA scaffolds, β-Ecdysone were each finely powdered and then mixed with KBr in a mass ratio of 1:100. This mixture was pressed into a transparent pellet and placed in an infrared spectrometer for analysis. The detection wavelength range was set between 500 and 4,000 cm⁻^1^, with 128 scans conducted at a resolution of 32 cm^−1^.

#### 2.3.6 *In vitro* degradation

The initial weight of a scaffold was noted as (m_0_). The scaffolds were immersed in 5 mL of FBS buffer and agitated at 60 rpm in a 37°C constant-temperature oscillation incubator. At specified time intervals, the scaffolds were retrieved and lyophilized, and the dry weight was recorded as (m_1_). The calculation formula is as follows:

$$\text{Degradation ratio}=\frac{m_{0}-m_{1}}{m_{0}}\times 100\%$$

#### 2.3.7 *In vitro* release

The drug-loaded scaffold was placed in a centrifuge tube containing 1 mL of FBS buffer and incubated at 37°C with oscillation at 60 rpm. At specified intervals, 100 μL of supernatant was collected, and an equal volume of fresh FBS solution was added to maintain the system under uniform conditions. The collected supernatant was analyzed using a UV spectrophotometer, with absorbance measured at 275 nm. The concentration of the drug was determined using a previously established standard curve. The drug release assay was conducted over 8 weeks, and the mean values were calculated from three parallel samples taken from each experimental group.

#### 2.3.8 Morphology of PLGA composite scaffolds

The morphology of the composite scaffolds was analyzed using scanning electron microscopy (SEM). Samples were prepared by attaching them directly to conductive adhesive and coating them with gold. Gold sputtering was carried out at a current of 10 mA for 45 s. After gold coating, the samples were imaged using SEM at an accelerating voltage of 3 kV.

### 2.4 Application of PLGA composite scaffolds *in vitro*

#### 2.4.1 Preparation of sample extraction solution

Following ISO 10993-12, two material extracts with different glucose concentrations were prepared. The PLGA composite scaffolds were added to serum-free L-DMEM medium (containing 5.5 mmol/L glucose) and H-DMEM medium (containing 25 mmol/L glucose) at a concentration of 0.2 g/mL. These were then placed in a 37°C constant-temperature oscillating incubator and shaken at 60 rpm for 72 h. The supernatant was subsequently collected to obtain the required sample extracts. Aseptic methods were strictly followed throughout the process.

#### 2.4.2 Cytotoxicity assay of PLGA composite scaffolds

Conventional isolation and culture of SD rat bone marrow stem cells (BMSCs). BMSCs were co-cultured with the extract, and the cytotoxicity of the PLGA composite scaffold was assessed using the CCK8 method. Third-generation BMSCs were seeded into 96-well plates at a density of 5,000 cells per well and divided into four groups: low-glucose blank group (L), high-glucose blank group (H), low-glucose scaffold group (L + CS), and high-glucose scaffold group (H + CS), with five replicate wells in each group. Once the cells adhered to the wells, L-DMEM and H-DMEM complete media were added to the low-glucose and high-glucose blank groups, respectively. Similarly, L-DMEM and H-DMEM complete media were added to the low-glucose and high-glucose scaffold groups. After 48 h of incubation, cell viability in each group was measured using the CCK8 method.

#### 2.4.3 Cytocompatibility testing of PLGA composite scaffolds

PLGA composite scaffolds were placed into 48-well plates and divided into two groups: the low-glucose scaffold group and the high-glucose scaffold group. To each scaffold, 100 μL of L-DMEM or H-DMEM complete medium containing a cell concentration of 1 × 10^4^ cells/mL was added. The plates were incubated for 4 h, after which an additional 200 μL of medium was added to each well, and incubation was continued. On days 3 and 7, the cells were stained with Live/Dead Staining Kit and observed for growth under a laser confocal microscope.

### 2.5 Application of β-E/PLGA composite scaffolds *in vivo*

Twenty-four SPF-grade male SD rats, aged 6–8 weeks, were obtained from the Laboratory of Experimental Animals at Guangxi Medical University in Nanning, China. The rats were randomly assigned to two groups: normal (N) and diabetic (DM), with 12 rats in each group. The diabetic model was established following a previously reported method (Tawulie et al., 2023). Briefly, the rats in the N group were fed a standard chow diet, while those in the DM group were given a high-sugar, high-fat diet for 4 weeks. After this period, the DM group received an intraperitoneal injection of 1% streptozotocin (STZ) at a dose of 30 mg/kg, whereas the N group received an injection of sodium citrate buffer at the same dose. Feeding continued for an additional 4 weeks, and fasting blood glucose was measured weekly from blood samples collected from the tail vein. A fasting blood glucose level of ≥11.1 mmol/L for 4 consecutive weeks was used as the criterion for the successful establishment of the diabetic model; rats that did not meet this criterion were excluded.

**FIGURE 2:**
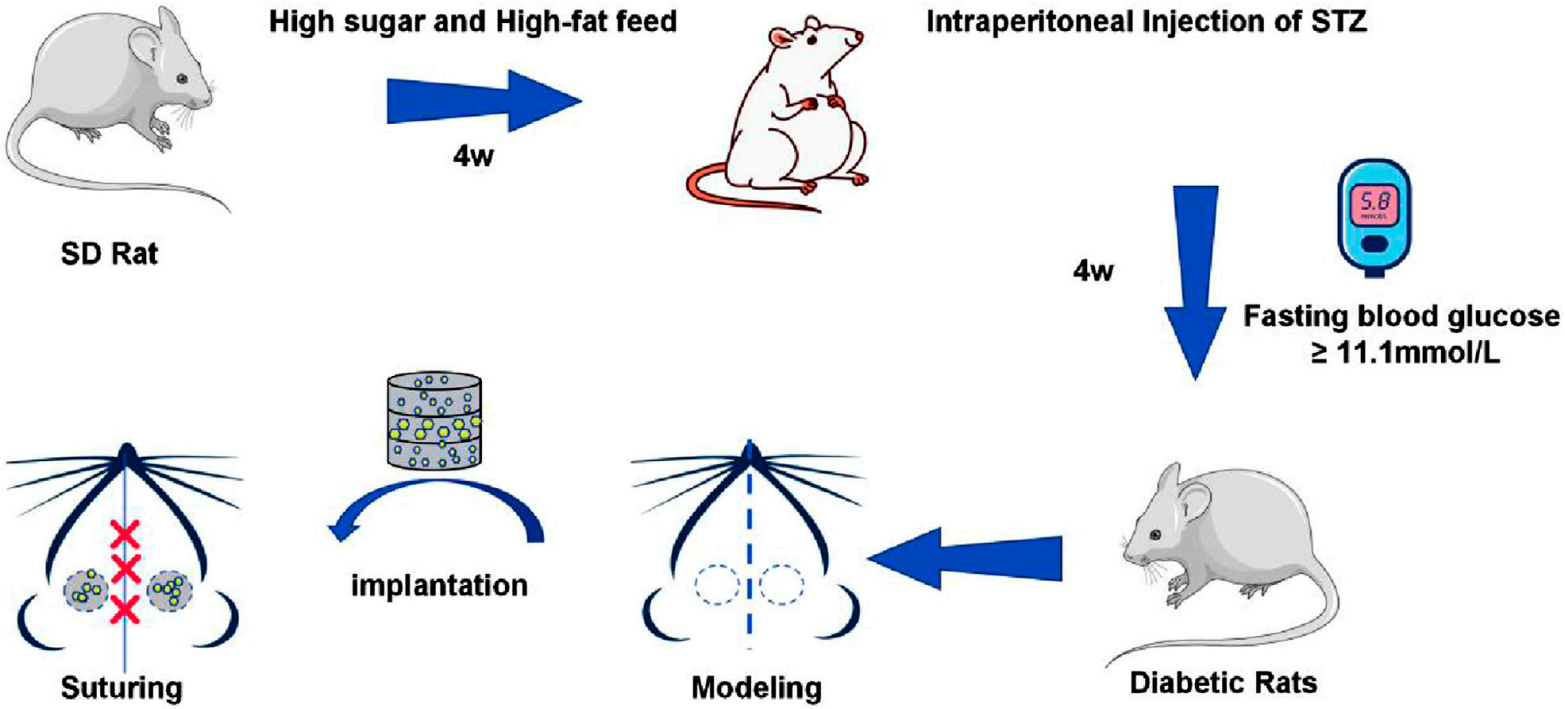
Flow chart of diabetic rats model and skull defect model.

The rats in the normal group were randomly divided into two subgroups: a blank group (Blank) and a normal control group (N + CS), with 6 rats in each. Similarly, the diabetic group was randomly divided into a PLGA composite scaffold group (DM + CS) and a β-E/PLGA composite scaffold group (DM + CS-βE), also with 6 rats per group. A cranial bone defect model was then constructed for all rats. After anesthetizing the rat with 2% sodium pentobarbital, place it in a prone position and make a 3 cm longitudinal incision along the skin of the cranial roof using a No. 11 surgical blade. Separate the skin, muscle, and periosteum layer by layer. Two 5 mm critical bone defects were created on both sides of the midline of the rat’s skull using a Lowspeed Handpiece (NSK, Japan) equipped with a 5 mm diameter deboning drill. PLGA composite scaffolds were implanted in the N + CS and DM + CS groups, β-E/PLGA composite scaffolds were implanted in the DM + CS-βE group, and no material was implanted in the Blank group. The skin and mucous membranes were tightly sutured, and penicillin was administered once daily for 3 days post-surgery (Figure 2). At 4 and 8 weeks postoperatively, the rats were euthanized by CO_2_, and the skulls were collected for micro-CT scanning, HE staining, Masson staining, and immunohistochemical staining. At 8 weeks, the hearts, livers, spleens, lungs, kidneys, and brains were also collected for HE staining.

### 2.6 Statistical analysis

SPSS 25.0 software and GraphPad Prism8 software were used to analyze and graph the data ( $\bar{x}\pm s$ ), and the one-way ANOVA method was used for analysis and comparison, while the two-by-two comparisons between groups were made using the LSD-t method, with the test criterion of α = 0.05, and the difference was considered to be statistically significant at *P* < 0.05.

## 3 Results and discussion

Diabetic patients are prone to bone metabolism disorders, leading to difficulty in recovering from bone resorption and, ultimately, the formation of bone defects. Large bone defects are often difficult to heal on their own, seriously damaging the patient’s quality of life and even leading to life-threatening conditions (Zhou et al., 2021). Currently, there is no definitive therapy or drug for treating diabetic bone defects, making this a key focus and challenge in medical research. β-Ecdysone, a phytohormone with multiple biological activities, is derived from a variety of plants and has a broad range of applications. It is widely available and has minimal toxic side effects on the human body, which has led to its use in industries such as aquaculture and cosmetics (Arif et al., 2022). However, due to its poor water solubility and low bioavailability, a slow-release delivery system is needed to enhance its effectiveness when administered topically. Tissue engineering scaffolds based on biomaterials show great application promise in the field of bone defect repair (Distler et al., 2020). PLGA, a widely studied biodegradable polymer, as well as a widely used biomaterial, has garnered significant attention, and materials based on PLGA have been approved by the FDA for various biomedical applications (Zhao et al., 2021). Numerous commercial products, such as absorbable sutures, drug delivery platforms, and bone replacement materials, are already available based on PLGA technology.

### 3.1 Characterization of PLGA scaffolds

As illustrated in Table 1, Composite scaffolds (S3) displayed a porosity of 82.19% ± 2.23%, a hydrophilic swelling rate of 926.56% ± 15.29%, and a drug loading efficiency of 2.32% ± 0.10%. These values fell between those of large aperture scaffolds (S1) and small aperture scaffolds (S2) across all three measurements. Pore size is crucial in bone formation, and research indicates that the optimal pore size for bone tissue regeneration ranges between 100 and 400 μm (Hollister, 2005; Murphy et al., 2010). During scaffold preparation, NaCl particles of two different sizes were screened: large particles (250–400 μm) and small particles (100–250 μm) were used to create suitable pore sizes for bone tissue regeneration (Lee et al., 2003). The porosity of the scaffold is recognized as crucial for enhancing cell proliferation and growth. Higher porosity facilitates cell migration and proliferation, providing ample surface area for interactions between cells and the scaffold while promoting the transport of oxygen and nutrients. However, if the porosity is too high, the scaffold may not be able to maintain optimum mechanical stiffness (Wei et al., 2009). Table 1 shows that S1 has the highest porosity, while S2 has the lowest, attributed to the increased size of the porogenic particles. Larger particles result in less PLGA content per unit volume of the scaffold, leading to larger pores upon dissolution of the porogens. However, this also results in lower mechanical strength. In contrast, S3 maintains sufficient mechanical strength while achieving high porosity. A high hydrophilic dissolution rate indicates good hydrophilicity, which enhances the rapid penetration of nutrients (Jin et al., 2021). The drug loading rate indicates the scaffold’s capacity to carry drugs. As shown in Table 1, scaffolds with greater porosity also revealed higher hydrophilic swelling and drug loading rates. This is because increased porosity allows for a greater volume of drug solution to be accommodated. In summary, S3 achieves high porosity, a substantial hydrophilic swelling rate, and an impressive drug loading rate, aligning with our expectations.

**TABLE 1:** Porosity, swelling ratio and drug loading of different PLGA scaffolds.

	Porosity (%)	Swelling ratio (%)	Drug loading (% w/w)
S1	93.63 ± 2.84^b^	1,104.41 ± 32.25^b^	2.54 ± 0.07^b^
S2	65.93 ± 4.27^a^	574.37 ± 20.86^a^	1.99 ± 0.14^a^
S3	82.19 ± 2.23	926.56 ± 15.29	2.32 ± 0.10

S3 is the control, ^a^ *p* < 0.05, ^b^ *p* < 0.05. S1, Large-pore-size scaffolds; S2, Small-pore-size scaffolds; S3, Composite scaffolds. Data are mean values (n = 3) ± SD.

**FIGURE 3:**
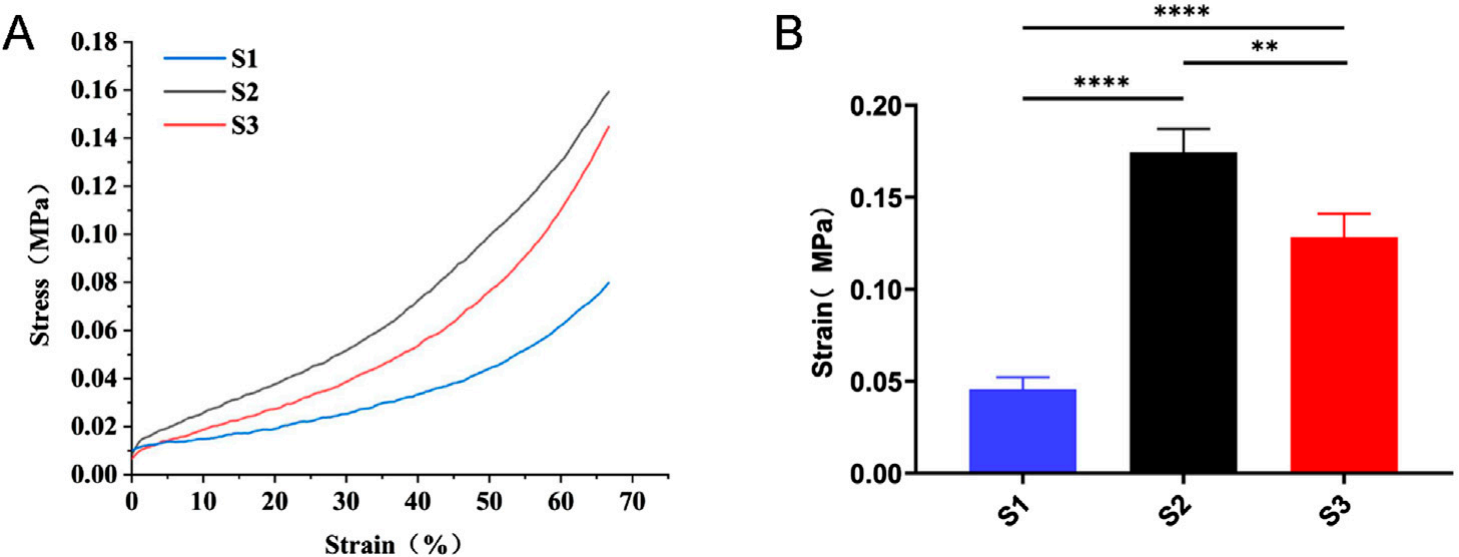
Compression strength testing of three types of scaffolds. **(A)** Typical stress-strain curves of S1, S2 and S3. **(B)** Compressive strength data of S1, S2, and S3. Data are mean values (n = 3) ± SD. **p < 0.01, ***p < 0.001.

The mechanical strength of a scaffold is crucial for withstanding pressure from surrounding soft tissues during bone tissue regeneration. A universal testing machine was utilized to assess the compression strength of the three scaffold types, as illustrated in Figure 3. The highest compression resistance was shown by S2, followed by S3, while the weakest compression resistance was demonstrated by S1. This suggests that S1’s low mechanical strength hampers its ability to maintain space in the bone defect area, which is detrimental to bone repair. In contrast, improved mechanical properties were shown by S3 and S2.

### 3.2 Degradation and release study

**FIGURE 4:**
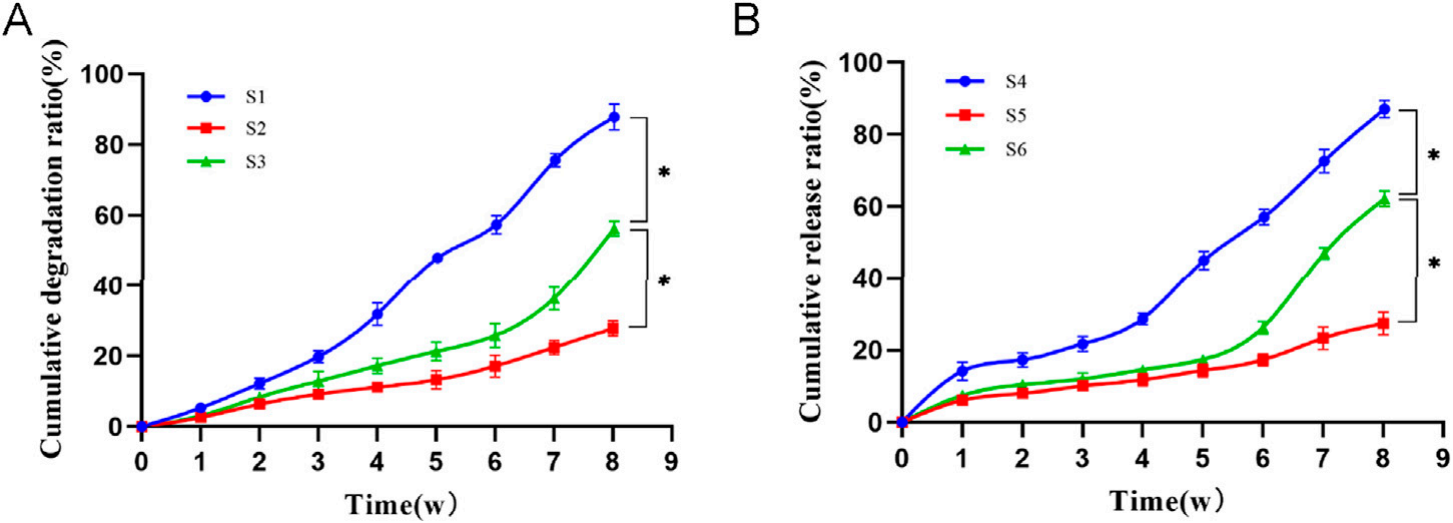
**(A)**: Cumulative degradation ratio of three types of scaffolds. Compared to S3, *p < 0.05. **(B)**: Cumulative drug release ratio of three types of scaffolds. S4: β-Ecdysone loaded large-pore-size scaffolds; S5: β-Ecdysone loaded small-pore-sizes; S6: β-E/PLGA composite scaffolds. Data are mean values (n = 3) ± SD. Compared to S6, *p < 0.05.

The results of the stent degradation experiments (Figure 4A) indicated that the degradation rate of S3 reached approximately 60% by week 8, while S1 had degraded nearly 90% and S2 showed only about 30% degradation. The findings from the drug release experiments (Figure 4B) demonstrated that the drug release rate is closely correlated with the scaffold degradation rate. S4 revealed a pronounced burst release effect, with nearly 20% of the drug released in the first week and a cumulative release of about 90% by week 8. This rapid release was attributed to S4’s large pore size and high porosity. In contrast, S5 released the drug slowly, with only about 20% released by week 8, while S6 achieved a cumulative release rate of around 60% by that same time. Previous studies have shown that the time required for bone defect repair is approximately 3 months (Saul and Khosla, 2022). The degradation time and drug release profile of PLGA composite scaffolds align well with the timeline of bone defect repair. This ensures that the scaffolding material provides consistent support in the bone defect area throughout the healing process while also degrading appropriately. PLGA composite scaffolds also facilitates prolonged drug release, contributing to sustained therapeutic efficacy.

### 3.3 FTIR spectroscopy

**FIGURE 5:**
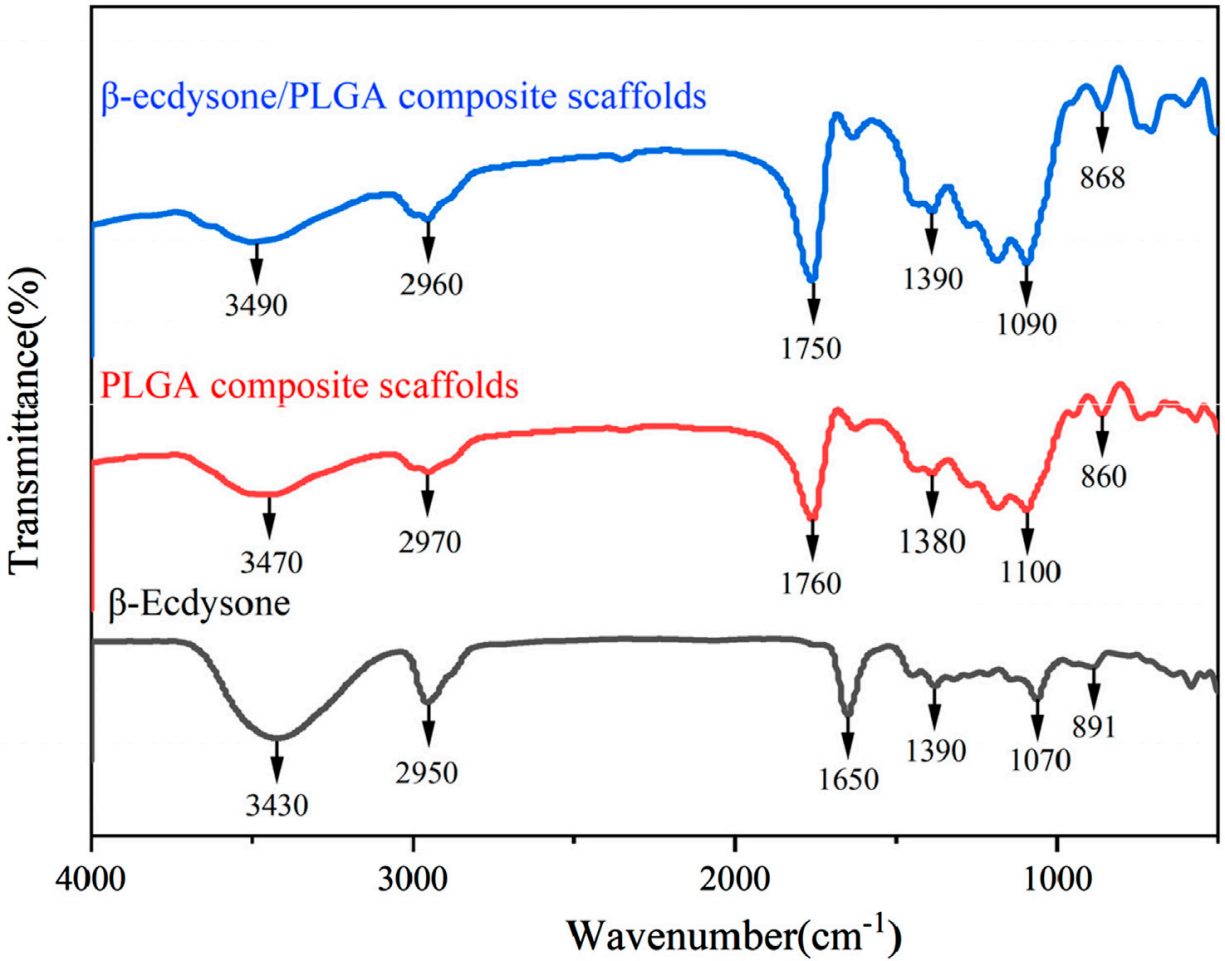
FTIR spectra of β-E/PLGA composite scaffolds, PLGA composite scaffolds and β-Ecdysone.

The FTIR spectra of the β-E/PLGA composite scaffolds, PLGA composite scaffolds, and β-Ecdysone are presented in Figure 5. The absorption peaks of the β-E/PLGA composite scaffolds largely aligned with those of the PLGA composite scaffolds; however, the absorption peak at 3,470 cm⁻^1^ for the blank scaffolds shifted to 3,490 cm⁻^1^ in the β-E/PLGA composite scaffolds. This shift suggests that the loading of the drug disrupts the hydrogen bonding system within the PLGA. In hydrogen bonding, the O-H bond length increases, leading to lower energy requirements for vibration and, consequently, absorption peaks at lower frequencies. Following the disruption of these hydrogen bonds, the peaks shift back to higher frequencies. Further, the absorption peak at 1,760 cm⁻^1^ shifted to 1,750 cm⁻^1^, likely due to overlap with the absorption peak at 1,650 cm⁻^1^ in the drug. Furthermore, the absorption peak at 1,100 cm⁻^1^ shifted to 1,090 cm⁻^1^, attributed to the stretching vibration of the alcohol C-O in the drug. These spectral changes indicate successful encapsulation of the drug, demonstrating improved encapsulation efficacy.

### 3.4 Morphology of PLGA composite scaffolds

**FIGURE 6:**
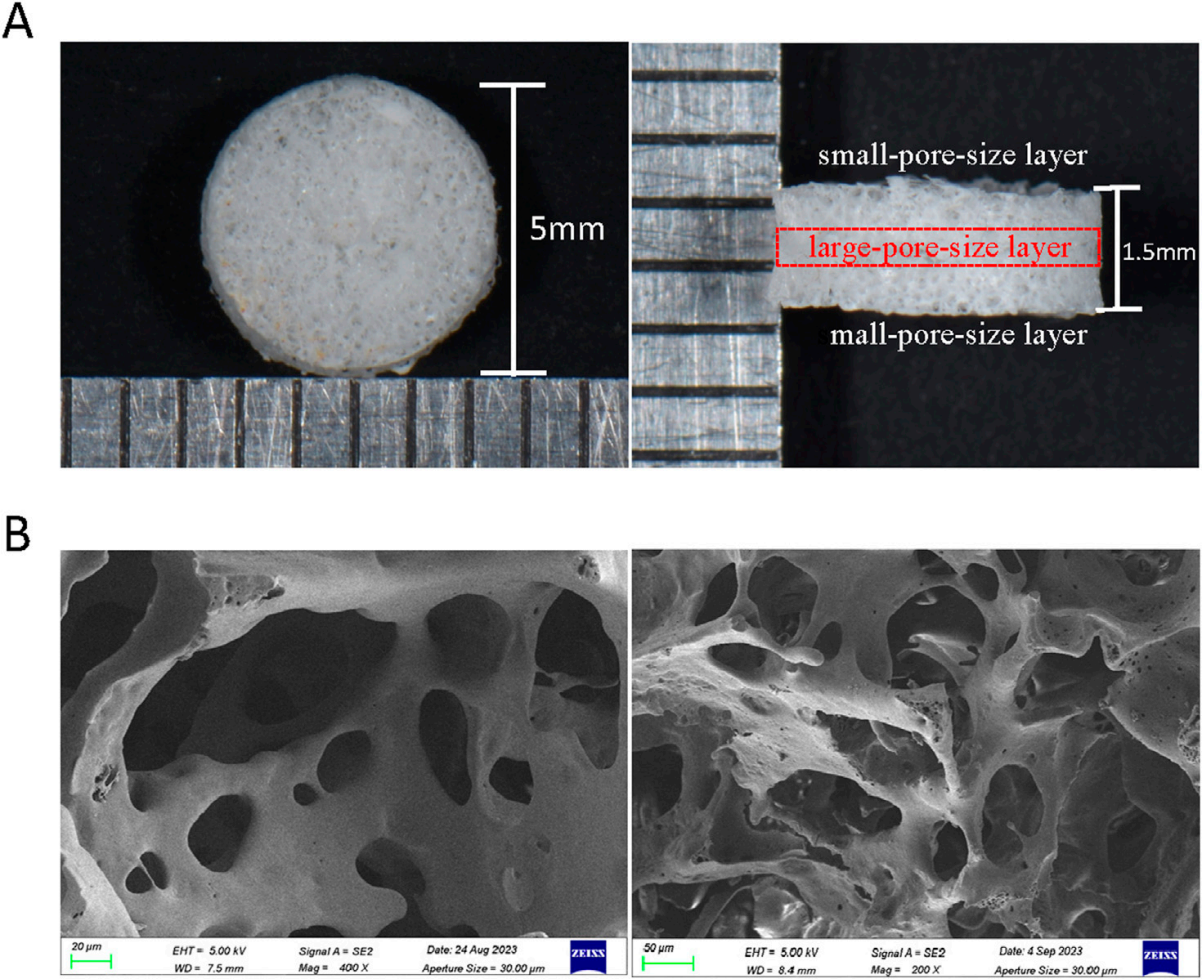
Morphology of PLGA composite scaffolds. **(A)** General diagram of PLGA composite scaffolds (side view and top view). **(B)** SEM of PLGA composite scaffold.

The bulk morphology and electron microscope images of composite scaffolds are displayed in Figure 6. Overall, a sandwich-like layered structure is characterized by a large pore size in the middle layer and smaller pores in the outer layers. Through scanning electron microscopy, it was demonstrated that the internal pores of the composite scaffold are interconnected and exhibit both large and small sizes, which is beneficial for drug loading and slow release.

### 3.5 Biosafety of PLGA composite scaffolds *in vitro*

**FIGURE 7:**
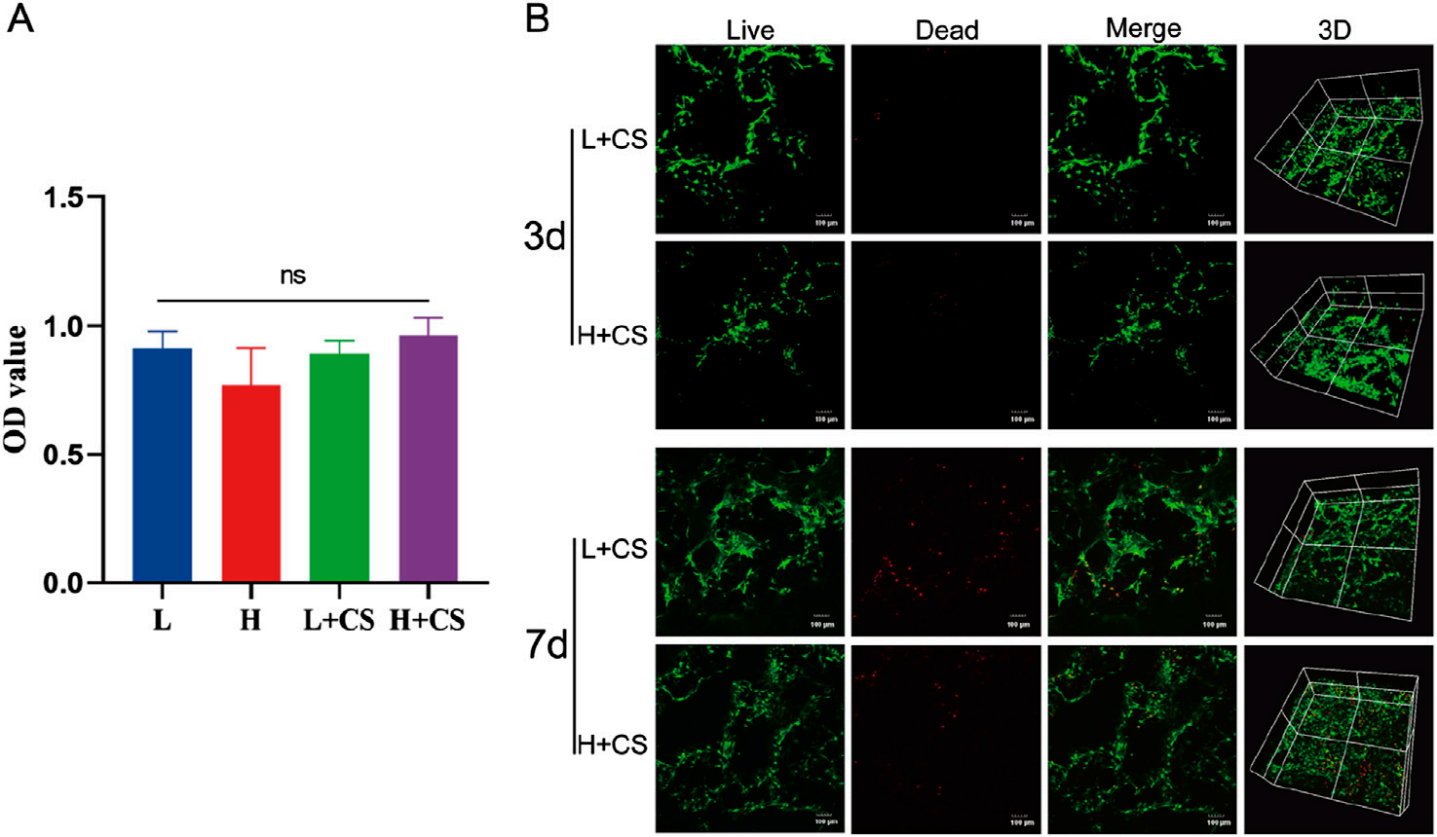
Biosafety analysis of PLGA composite scaffolds. **(A)** CCK8 assay showing the cell viability of BMSCs incubated with different sample extraction solutions at 48 h. **(B)** Live/Dead staining of the BMSCs at 3 and 7 days (green colors indicate living cells). Data represents mean ± SD (n = 3).

Next, the *in vitro* biosafety of the PLGA composite scaffolds was tested. The identification results of BMSCs were shown in Supplementary Figure S1. The results of the CCK8 assay (Figure 7A) indicated that the extracts of the composite scaffolds displayed no toxicity to rat bone marrow MSCs when compared to the blank control group, in both low and high-glucose media. The results of the live-dead cell staining assay (Figure 7B) demonstrated that the bone marrow MSCs survived and proliferated on the composite scaffolds in both low and high-glucose environments. These findings suggest that the composite scaffolds possess reliable *in vitro* safety and cytocompatibility.

In summary, the PLGA composite scaffold was successfully developed that demonstrates high porosity, a significant hydrophilic swelling rate, an effective drug loading capacity, good mechanical properties, and an appropriate degradation time along with favorable slow-release characteristics. In addition, the PLGA composite scaffold also had good biosafety *in vitro*, making it suitable for further animal experiments.

### 3.6 Application of β-E/PLGA composite scaffolds *in vivo*

The animal experiments complied with the ethical requirements of Guangxi Medical University Laboratory Animal Center. To evaluate the effect of drug-carrying composite scaffolds in the treatment of diabetic bone defects, a diabetic rat cranial bone defect model was constructed. Based on the previously established criteria for the establishment of a diabetic rat model (Sun et al., 2022), a successful diabetic model is indicated when fasting blood glucose levels are maintained at ≥11.1 mmol/L for 3–4 weeks. The results (Table 2) showed that the fasting blood glucose levels of the rats in the diabetic group consistently exceeded 11.1 mmol/L for the entire duration of 4 weeks. The weight of the rats in the diabetic group continued to decline compared to their pre-modeling weights, which aligns with the typical symptoms associated with diabetes mellitus (Barragán-Bonilla et al., 2019).

**TABLE 2:** Weight and FBG data of rats at different times.

Time(w)	N		DM	
	Weight(g)	FBG (mmol/L)	Weight(g)	FBG (mmol/L)
0	351.48 ± 16.17	4.19 ± 0.30	417.18 ± 18.82	4.33 ± 0.28
1	397.57 ± 21.29	4.44 ± 0.38	432.98 ± 20.65	15.91 ± 1.83
2	430.36 ± 24.59	4.45 ± 0.39	426.73 ± 17.63	15.57 ± 2.14
3	449.92 ± 20.52	4.25 ± 0.41	409.88 ± 15.22	16.94 ± 1.79
4	466.80 ± 18.49	4.32 ± 0.50	381.13 ± 14.71	17.50 ± 2.29

N, normal group; DM, diabetes group; FBG, fasting blood glucose.

**FIGURE 8:**
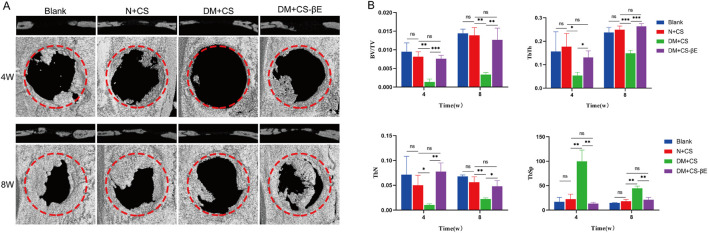
β-E/PLGA composite scaffolds promoted skull defect healing at 4 and 8 weeks. **(A)** Micro-CT images of bone defects at 4 and 8 weeks. **(B)** BV/TV, TbN, TbTh and TbSp analysis of the bone defects at 4 and 8 weeks. *p < 0.05, **p < 0.01, ***p < 0.001.

The rat extreme bone defect model is widely recognized for evaluating bone repair in tissue engineering. In this study, extreme bone defects with a diameter of 5 mm were created bilaterally in the skulls of the rats. To assess bone regeneration in the defect areas post-surgery, rat skulls were collected at 4 and 8 weeks for micro-CT scanning. The results (Figure 8A) indicated that new bone formation was significantly reduced in the DM + CS group compared to the N + CS group. Conversely, the DM + CS-βE group exhibited significantly greater new bone formation than the control group.

Bone volume fraction (BV/TV) serves as an indicator of bone quantity in the area of interest, while the number of trabeculae (TbN), trabecular thickness (TbTh), and trabecular separation (TbSp) are key metrics for evaluating the spatial morphology and structure of trabecular bone. High values of BV/TV, TbN, and TbTh indicate substantial new bone formation, while a high TbSp value reflects greater distances between trabeculae and suggests poor bone quality. The findings (Figure 8B) revealed that the DM + CS group had lower BV/TV, TbN, and TbTh, along with higher TbSp compared to the N + CS group. This suggests that the DM + CS group had a reduced amount of new bone and poor bone quality, indicating that the diabetic condition hindered bone defect repair. In contrast, the DM + CS-βE group showed higher BV/TV, TbN, TbTh, and lower TbSp compared to the DM + CS group, indicating that β-Ecdysone enhanced the repair of cranial bone defects in a diabetic model.

**FIGURE 9:**
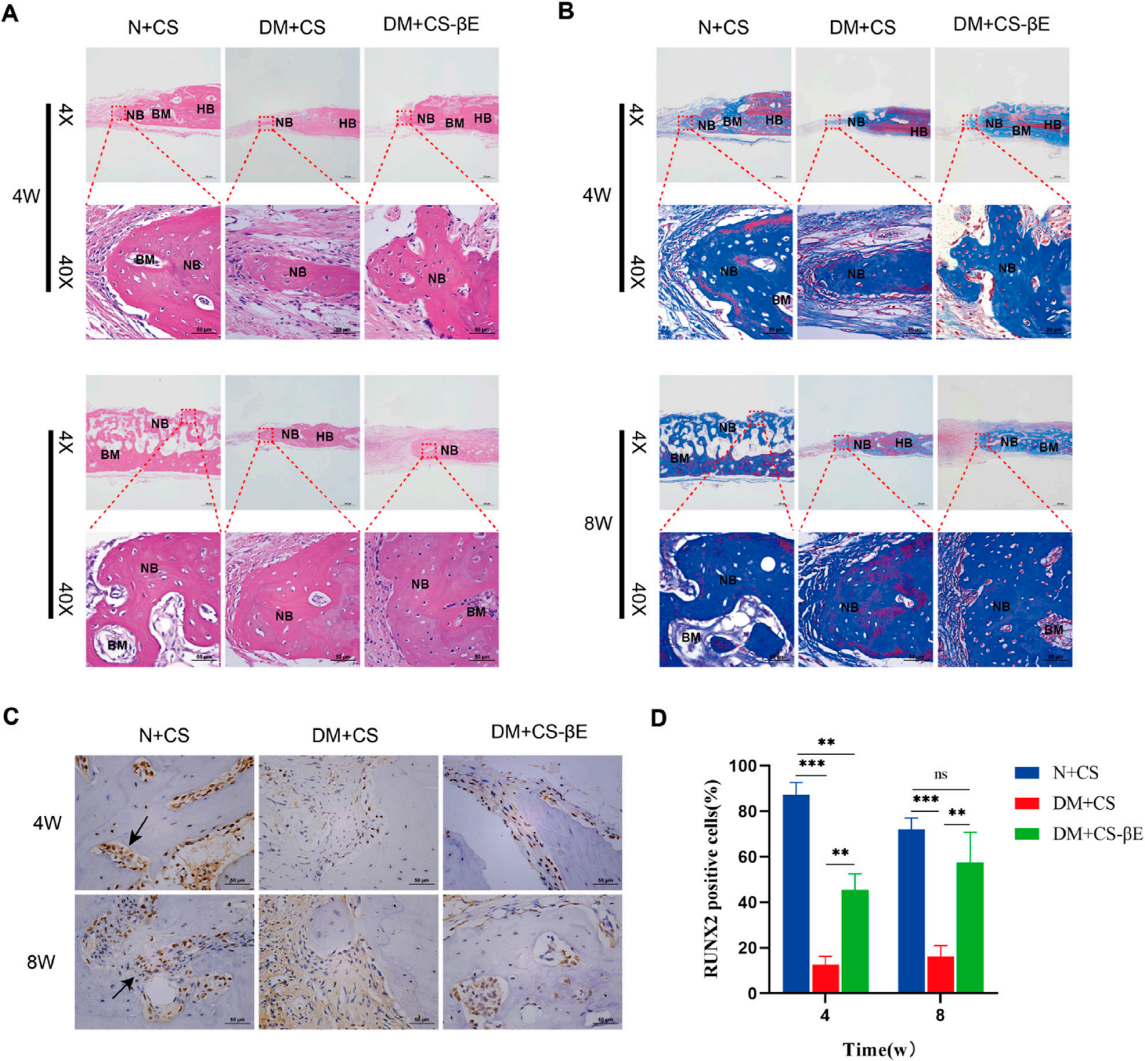
Histological evaluation of bone regeneration at 4 and 8 weeks. **(A)** H&E staining at 4 and 8 weeks (×200, ×400). **(B)** Masson-trichrome staining at 4 and 8 weeks (×200, ×400) (HB, host bone; NB, regenerated bone; BM, bone marrow). **(C)** Immumohistochemical staining for detecting the expression of Runx 2 at 4 and 8 weeks (×400) (Black arrows indicate RUNX2 positive cell in new bone tissue). **(D)** Immumohistochemical staining analysis. **p < 0.01, ***p < 0.001.

To further validate the hypothesis, H&E staining, Masson staining, and immunohistochemical staining of bone tissues from the N + CS, DM + CS, and DM + CS-βE groups was carried out. These analyses provided additional insights into bone tissue regeneration. The H&E staining results (Figure 9A) revealed sparse vascularization in most of the new bone regions in the DM + CS group at both 4 and 8 weeks, indicating that osteogenesis was somewhat inhibited. In contrast, the N + CS and DM + CS-βE groups exhibited a significant presence of new blood vessels in the bone regions, suggesting active osteogenesis. While the amount of neovascularization in the N + CS group was slightly greater than in the DM + CS-βE group, the inclusion of β-Ecdysone contributed to improved bone regeneration in the defect areas of diabetic rats. Masson staining results (Figure 9B) corroborated the findings from the H&E staining, showing more neovascularized bone tissue and abundant blood vessels in the N + CS and DM + CS-βE groups. Conversely, the DM + CS group displayed low levels of neovascularization and sparse vascularization. RUNX2, a member of the RUNX family of transcription factors, plays a crucial role in osteoblast differentiation and bone morphogenesis and is specifically expressed in osteoblasts, where it is localized in the nucleus (Komori, 2019). Immunohistochemical staining results (Figures 9C, D) showed that RUNX2 protein expression was significantly higher in the N + CS group compared to the DM + CS group (indicated in blue). Similarly, RUNX2 protein expression was substantially higher in the DM + CS-βE group than in the DM + CS group. These findings suggest that β-Ecdysone mitigates the inhibitory effects of diabetes on bone defect repair and promotes the healing of diabetic bone defects.

**FIGURE 10:**
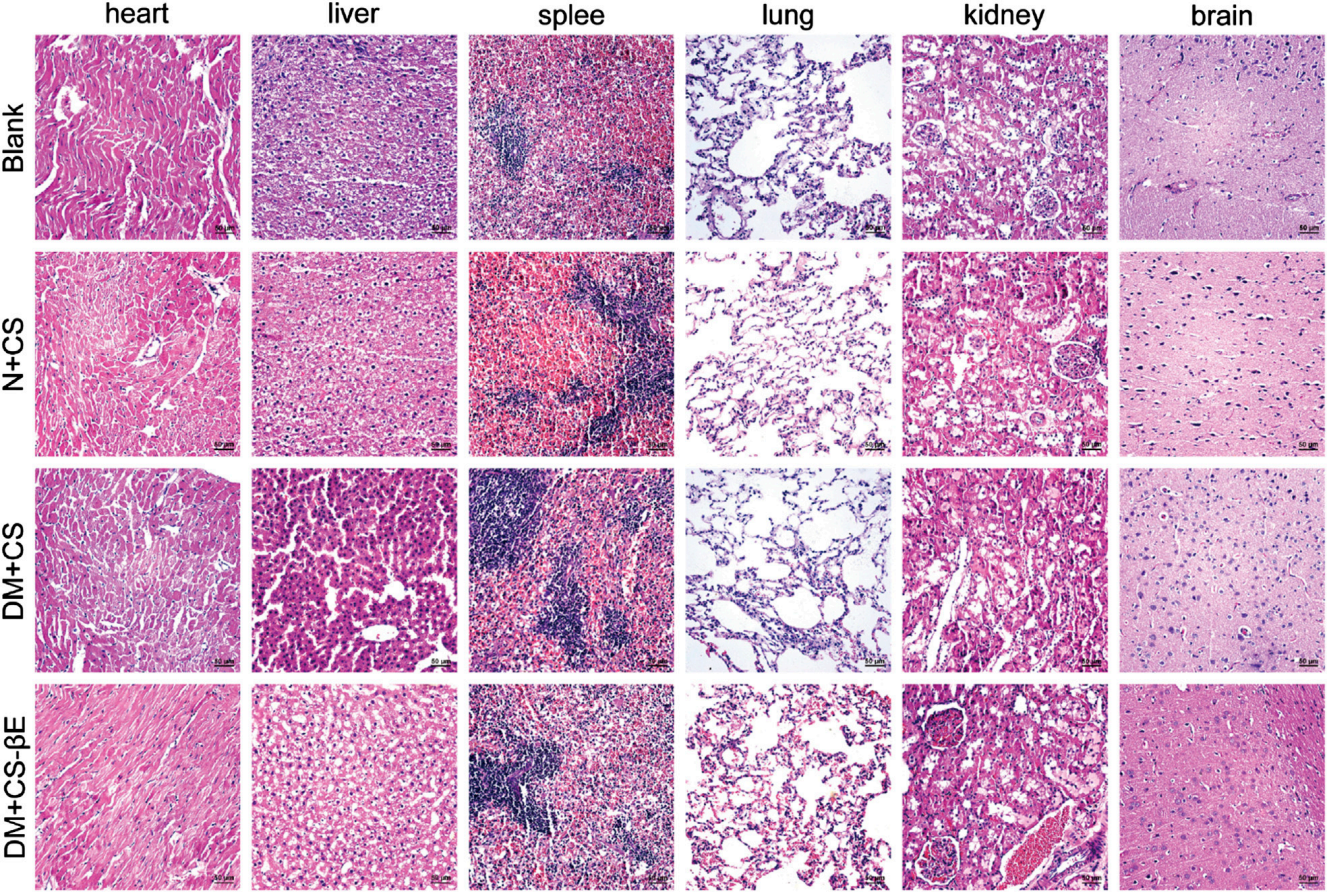
H&E staining of heart, liver, spleen, lung, kidney, and brain at 8 weeks (×200).

The H&E staining results of heart, liver, spleen, lung, kidney, and brain tissues from rats in each group at 4 weeks (Supplementary Figure S2) and 8 weeks (Figure 10) were compared. The visceral tissues of rats in the N + CS, DM + CS, and DM + CS-βE groups did not exhibit any significant pathological changes compared to the Blank group. This indicated that the composite scaffolds did not cause any toxic damage to the vital organs *in vivo*, thereby confirming the *in vivo* safety of the composite scaffolds.

## 4 Conclusion

In this study, a PLGA composite scaffold with different pore sizes was successfully developed, which possessed excellent physical properties and biosafety. The composite scaffold combined with β-ecdysone can achieved slow release *in vitro*, which contributed to improve the bioavailability of β-ecdysone. In the diabetic rat cranial bone defect model, the implantation of the β-E/PLGA composite scaffold resulted in significant bone regeneration. The findings indicated that the PLGA composite scaffold effectively preserved the drug’s activity while ensuring reliable biosafety, thereby markedly enhancing the healing of cranial bone defects in diabetic rats. In conclusion, these results present a promising approach for the treatment of diabetes-related bone defects.

## Data Availability

The raw data supporting the conclusions of this article will be made available by the authors, without undue reservation.
