# Supplementary material for: β-ecdysone/PLGA composite scaffolds promote skull defect healing in diabetic rat
Source: Front Bioeng Biotechnol. 2025 Jan 13;12:1536102. doi: 10.3389/fbioe.2024.1536102 (PMC11770018; doi:10.3389/fbioe.2024.1536102)
Supplement: Supplementary file 1 [file DataSheet1.docx]

**β-Ecdysone/PLGA composite scaffolds promote skull defect healing in diabetic rat**

Yicai Luo^1†^, Ziwei Wu^1†^, Yingjuan Zhang^1^, Yang Qiao^1^, Yinge Wei^1^, Xuan Yan^1^, Xiangyu Ma^1^, Xianxian Huang^1^,Xiaoxia Zhong^1^,Zhimao Ye^1^,Xinping Lu^1^. Hongbing Liao^1*^

^1^ Guangxi Key Laboratory of Oral and Maxillofacial Rehabilitation and Reconstruction, College & Hospital of Stomatology, Guangxi Medical University, No.10 Shuangyong Road Nanning, Guangxi 530021, China; luoyicai1987@163.com (Y.L.);18772851152@163.com (Z.W.)

^*^ Correspondence:hongbing_liao@gxmu.edu.cn

^†^ These authors contributed equally to this work.

Conventional isolated and cultured of SD rat bone marrow stem cells(BMSCs), The third generation BMSCs were taken for identification, and the identification methods included osteogenic induction, adipogenic induction, chondrogenic induction and flow cytometry. After 14 days of osteogenic induction, BMSCs were stained with alizarin red staining, and calcium nodules were observed (orange-red). After 21 days of adipogenic induction, oil red O staining was performed, and lipid droplets were observed in the cells (orange-red). After 30 days of chondrogenic induction, alcian blue staining was performed, and cartilage nodules were observed (blue). Flow cytometry results showed that CD29 and CD90 antibodies were positively expressed on the cell surface, and CD31 and CD45 antibodies were negatively expressed, which was consistent with the characteristics of BMSC. The identification results were shown in Figure S1.

A B C


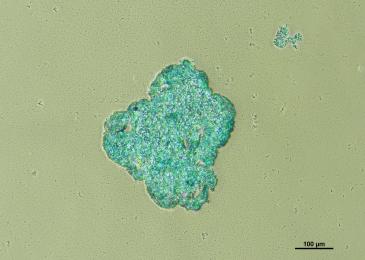

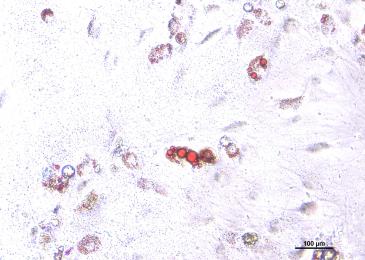

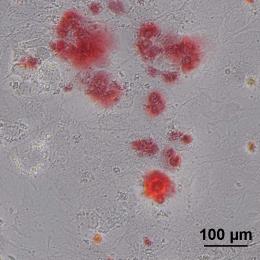


D


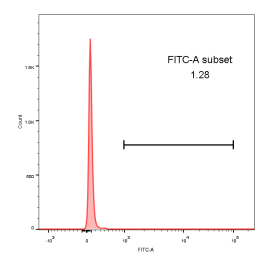

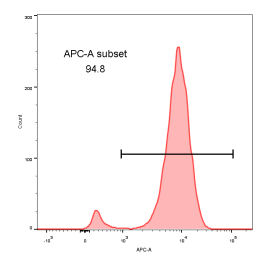

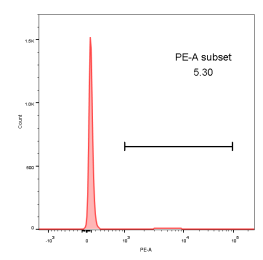

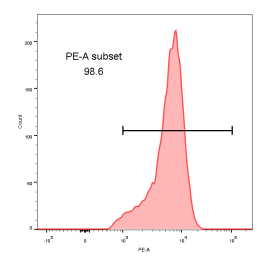


CD29

CD90

CD31

CD45

**Fig S1.** **(A)** Alizarin red staining. **(B)** Oil red O staining. **(C)** Alcian blue staining. **(D)** Flow cytometry: CD29(+), CD90(+) ,CD31(-) , CD45(-).

**Fig S2.** H&E staining of heart, liver, spleen, lung, kidney, and brain at 4 weeks (200×).

heart

liver

spleen

lung

kidney

brain


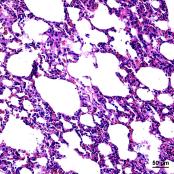

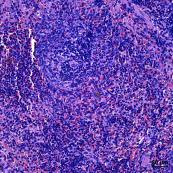

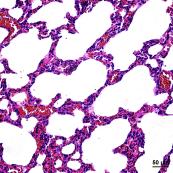

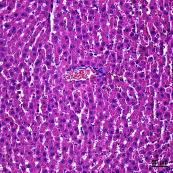

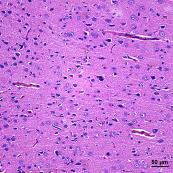

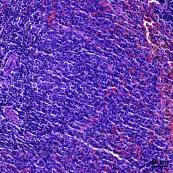

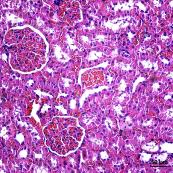

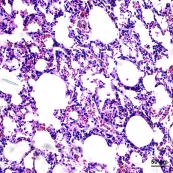

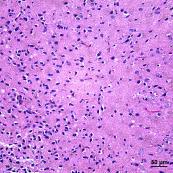

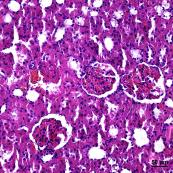

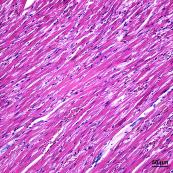

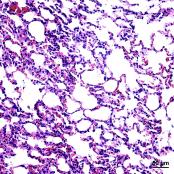

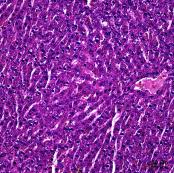

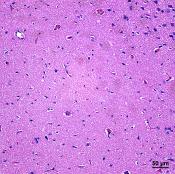

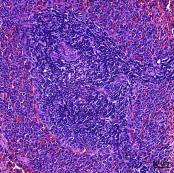

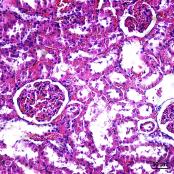

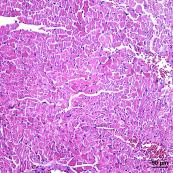

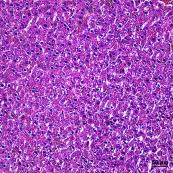

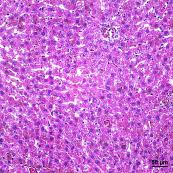

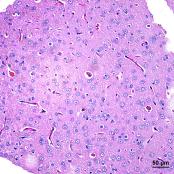

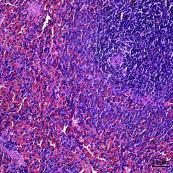

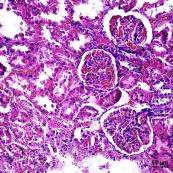

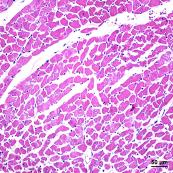

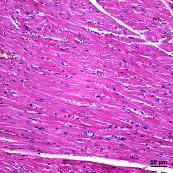


Blank

DM+CS

DM+CS-βE

N+CS
